# Correlation between plaque vulnerability of aorta and coronary artery: an evaluation of plaque activity by direct visualization with angioscopy

**DOI:** 10.1007/s10554-015-0669-z

**Published:** 2015-04-28

**Authors:** Jun Aono, Shuntaro Ikeda, Yuriko Katsumata, Haruhiko Higashi, Kousei Ohshima, Ken Ishibashi, Hiroshi Matsuoka, Kouki Watanabe, Mareomi Hamada

**Affiliations:** Division of Cardiology, Kitaishikai Hospital, Tokunomori, Ozu, Ehime Japan; Saha Cardiovascular Research Center, University of Kentucky, Lexington, KY USA; Division of Cardiology, Uwajima City Hospital, 1-1 Gotenmachi, Uwajima, Ehime 7988510 Japan; Department of Biostatistics, University of Kentucky, Lexington, KY USA; Division of Cardiology, Prefectural Niihama Hospital, Niihama, Ehime Japan; Department of Cardiovascular Medicine, Hiroshima University Graduate School of Biomedical and Health Sciences, Hiroshima, Japan; Division of Cardiology, Prefectural Imabari Hospital, Imabari, Ehime Japan; Division of Cardiology, Saiseikai Matsuyama Hospital, Matsuyama, Ehime Japan

**Keywords:** Atherosclerosis, Angioscopy, Thoracic aorta, Coronary artery, Atherosclerotic plaque

## Abstract

This study investigated the relationship between the degree of atherosclerotic changes in the descending thoracic aorta (TA) and the coronary artery using angioscopy. Twenty-five consecutive patients undergoing angioscopy of the TA and coronary angiography were enrolled in this study. Participants were divided into three groups according to the angioscopic grading of the TA: white plaque group (W-group), yellow plaque group (Y-group) and intensive yellow, ruptured plaque with ulceration and/or thrombus group (RP-group). The maximum plaque grade, plaque score, number of yellow plaques, frequency of yellow-plaque grades by coronary angioscopy, and SYNTAX score by coronary angiography were evaluated. Brachial-artery pulse wave velocity and high-sensitivity C-reactive protein level tended to be higher in the RP-group than in the other groups, although the differences were not statistically significant. The SYNTAX score was significantly higher in the RP-group than in the W-group (W-group 4.0 ± 3.6 vs. RP-group 17.5 ± 10.0, *P* = 0.045). In addition, the angioscopic maximum plaque grade, plaque score, and number of yellow plaques in the RP-group were significantly higher than in the W-group (maximum plaque grade W-group 0.8 ± 0.4 vs. RP-group 1.8 ± 0.8, *P* = 0.026; plaque score W-group 1.0 ± 1.2 vs. RP-group 4.0 ± 1.4, *P* = 0.014; and number of yellow plaques W-group 1.0 ± 1.2 vs. RP-group 2.5 ± 0.5, *P* = 0.023). The yellow-plaque grade in the coronary artery was correlated significantly with the plaque grading of TA (*P* = 0.043). Our study suggests that the angioscopic progression of aortic atherosclerosis is closely associated with vulnerability to and the extent of coronary stenosis, indicating that vulnerability toward atherosclerotic plaque development occurs simultaneously in the coronary tree and systemic arteries.

**Electronic supplementary material:**

The online version of this article (doi:10.1007/s10554-015-0669-z) contains supplementary material, which is available to authorized users.

## Introduction

Atherosclerotic plaque rupture and subsequent thrombus formation in the culprit lesion are recognized as pivotal events in the majority of cases of acute coronary syndrome.

Coronary angioscopy is a powerful tool for evaluating atherosclerosis of the coronary artery by direct visualization of intracoronary surface morphology, which provides detailed information about plaque vulnerability that other modalities cannot detect [1–4]. In angioscopy, yellow color intensity of plaque and thrombus formation is determined by the thickness of the fibrous cap and is associated with plaque vulnerability [5–7]. In particular, angioscopic evaluation can illuminate the presence of a thrombus or endothelial irregularities such as ulceration, fissures, or tears. This directly visualized information about the detailed vascular surface morphology, including color, cannot be detected by other imaging methods such as computed tomography (CT), magnetic resonance imaging (MRI), or ultrasonography.

Atherosclerosis is widely recognized as a systemic inflammatory disease that is often manifested by patients with polyvascular disease [8, 9]. The presence of carotid plaques has been associated with an increased risk of cardiovascular events in patients with coronary artery disease (CAD) [10]. In addition, recent reports have shown that there is a significant relationship between atherosclerotic changes in the thoracic aorta (TA), common carotid intima-media thickness (IMT), and the angiographic extent of coronary artery stenosis in patients with severe CAD [11]. The IMT of the TA also correlates with coronary atherosclerosis [12].

However, the relationship of angioscopic plaque vulnerability between the TA and the coronary artery has not yet been elucidated. The purpose of this study was to determine the correlation between plaque vulnerability in the TA and the coronary artery via angioscopic analysis.

## Methods

### Study patients and protocol

**Fig. 1:**
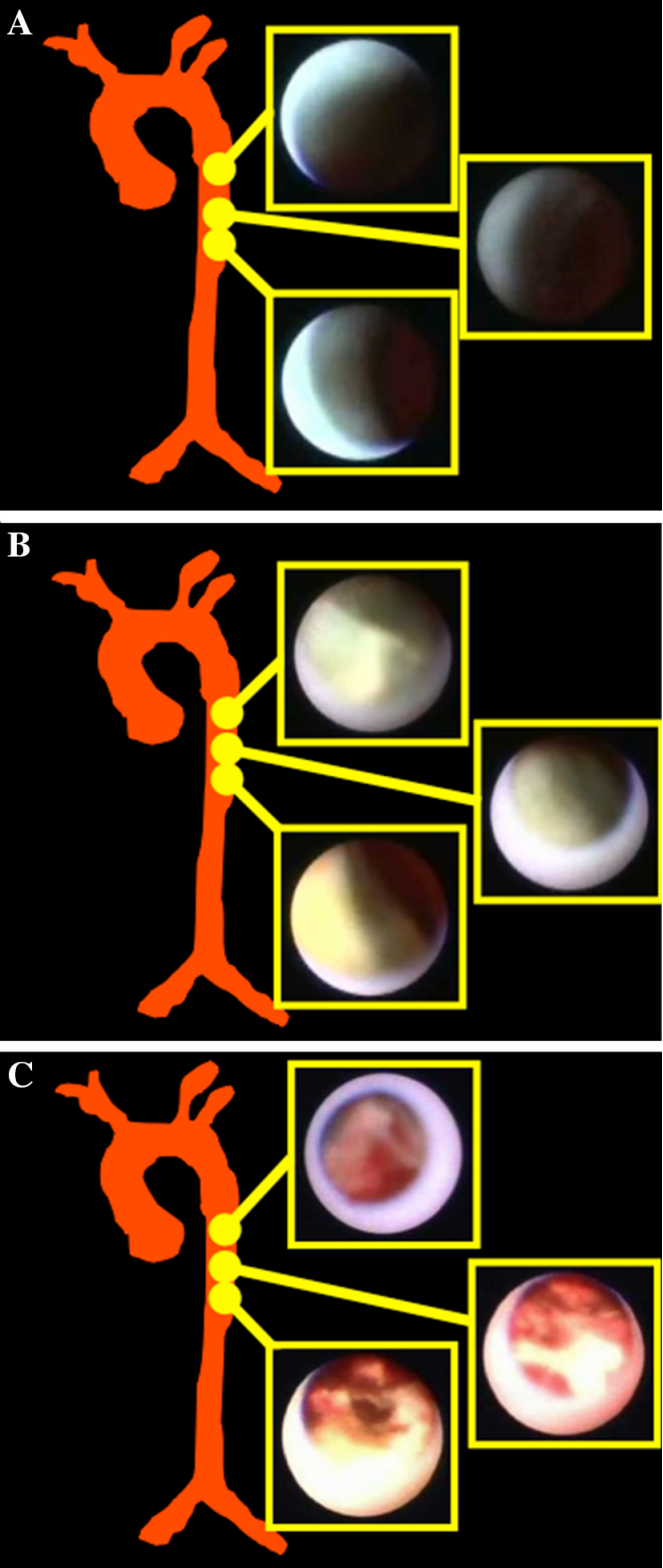
Representative angioscopic findings of the vessel surface of the thoracic aorta (TA) in the W-group (W), Y-group (Y), and RP-group (RP). **A** No yellow plaque or smooth vascular surface is seen in the TA (W). **B** Slightly yellow plaque and smooth vascular surface are detected in the TA (Y). **C** Intensive yellow, ruptured plaque with ulceration and/or thrombus and irregular vascular surface are observed in the TA (RP)

Between June 2006 and January 2007, 25 consecutive patients suffering from angina who underwent both coronary angiography (or angioplasty) and angioscopy via a femoral arterial approach were enrolled in this study. In patients who underwent percutaneous coronary intervention, we observed non-culprit vessels. Exclusion criteria included left main trunk disease, tortuous vessels with expected difficulty in advancing coronary angioscopy, congestive heart failure, renal insufficiency with baseline creatinine >1.5 mg/dL, or lack of informed consent. The participants were divided into three groups according to the following angioscopic grading of TA: white plaque group (W-group), yellow plaque group (Y-group), and intensive yellow, ruptured plaque with ulceration and/or thrombus group (RP-group) (Fig. 1, W-group: Video S1, Y-group: Video S2, RP-group: Video S3 and Video S4). The maximum plaque grade, plaque score, number of yellow plaques within a coronary artery, and frequency of the yellow-plaque grades were evaluated by coronary angioscopy in each group. In addition, SYNTAX score by coronary angiography was evaluated to determine the relationship between angioscopic plaque vulnerability of the TA and the severity of CAD (details below) [13]. Brachial-artery pulse wave velocity (ba-PWV) and serum high-sensitivity C-reactive protein (hs-CRP) level were also evaluated to investigate whether the angioscopic findings of the TA correlated with arterial stiffness and systemic inflammation (details below). The prevalence of hypertension, hypercholesterolemia, smoking habit, and diabetes was evaluated. Patients with hypertension were those with blood pressure >140/90 mm Hg or those receiving antihypertensive drugs. Patients with diabetes were identified as those with a fasting blood glucose >126 mg/dL, glycated hemoglobin (HbA1c) level >6.5 %, or those receiving drugs or insulin therapy for diabetes mellitus. Patients with dyslipidemia were identified as those with a total cholesterol level >220 mg/dL, low-density lipoprotein cholesterol level >140 mg/dL, high-density lipoprotein cholesterol <40 mg/dL, triglyceride levels >150 mg/dL, or those receiving drugs for dyslipidemia.

### Angioscopic equipment and procedures

Catheterization was performed via the femoral artery using a 6- or 7-F sheath and catheter. Heparin (5000 IU) was administered and 2.5–5 mg isosorbide dinitrate was injected into the coronary artery before the procedure. In patients who underwent percutaneous coronary intervention, 10,000 IU of heparin was administered at the beginning of the procedure. An angioscope MC-800E (Nihon Kohden, Tokyo, Japan) and fiber optic AS-003 were used for angioscopy. A 4-F over-the-wire catheter (Medikit Co., Ltd. Tokyo, Japan) for angioscopic observation was inserted into the coronary artery over the guide wire to the desired position. The probing catheter system was comprised of an outer catheter and an inner catheter. After removing the inner catheter with the guide wire, an angioscope was advanced through the probing outer catheter to the tip. Angioscopic observations were performed while blood was cleared from the view by the manual injection of 3 % dextran-40 (a very gentle manual injection of 2–4 mL/s). The whole descending TA was continuously studied and the existence of yellow plaques and thrombi were evaluated.

### Definition and analysis of angioscopic findings

Angioscopic definitions were defined according to previous reports of coronary angioscopy [2, 6]. Plaque grade was defined as 0 (white), 1 (light yellow), 2 (yellow), or 3 (intensive yellow). Plaque score was defined as the sum of the plaque grade within a coronary artery. Maximum plaque grade was defined as the highest yellow plaque grade within a coronary artery by angioscopic findings. In order to investigate the relationship between plaque surface characteristics of the coronary artery and the TA, we examined angioscopic findings of the coronary artery including the maximum plaque grade, plaque score, number of yellow plaques, and frequency of the yellow-plaque grades in each group.

### SYNTAX score and angiography

Coronary artery stenosis was defined as stenosis with a diameter ≥50 %. On the basis of the baseline diagnostic coronary angiogram, each coronary lesion producing stenosis with a diameter ≥50 % in vessels ≥1.5 mm in diameter was separately scored, and these scores were combined to provide the overall SYNTAX score, which was calculated using the SYNTAX score calculator Ver 2.11 (available on the SYNTAX website). The SYNTAX scores were independently evaluated by 2 experienced interventional cardiologists who were blinded to the laboratory data and angioscopic findings.

### Brachial-artery pulse wave velocity and serum high-sensitivity C-reactive protein level

Systemic arterial stiffness was evaluated by ba-PWV using Form PWV/ABI™ (Japan COLIN, Tokyo Japan) at 25 °C, following 10-min bed rest. Utilized as a marker for systemic inflammation, serum hs-CRP level was measured using the latex-enhanced immunoturbidimetric assay (Roche diagnostics, Japan), with detection limit <0.01 mg/dL.

### Statistical analysis

The differences between groups were compared using one-way analysis of variance (ANOVA) followed by a post hoc Tukey–Kramer test. Data for the plaque score, maximum plaque grade, number of yellow plaques, and SYNTAX score did not show normal distributions, so these scores were compared using Kruskal–Wallis non-parametric ANOVA followed by Dunn’s post hoc pairwise test. Normality was confirmed by visual inspection of the distribution and Q–Q plots. To determine the frequency of the each yellow-plaque grade, each of the plaques was counted according to the yellow-plaque grade in each group. The Mantel–Haenszel exact test was used to evaluate the correlation between the angioscopic grading of TA and the yellow-plaque grade within a coronary artery, both of which were considered to be on an ordinal scale. Categorical variables were compared using Fisher’s exact test. A value of *P* < 0.05 was considered statistically significant. Statistical analyses were performed with SPSS version 22 (SPSS, IBM, Armonk, NY, USA) and SAS version 9.3 (SAS Institute, Cary, NC, USA).

## Results

### Baseline patient characteristics

Optimal images were obtained with both aortic and coronary angioscopy in the 25 patients. Table 1 shows the clinical characteristics of the study participants, including 5 patients in the W-group, 14 patients in the Y-group, and 6 patients in the RP-group. The prevalence of hypertension, hypercholesterolemia, or current smoking was similar between the groups. In addition, lipid levels and blood pressure were not statistically different among the groups. On the other hand, the prevalence of diabetes mellitus was markedly higher in the RP-group than in the other groups. HbA1c levels in the RP-group were significantly higher than those in the other groups (*P* < 0.01; Table 1).

**Table 1:** Patient characteristics

	White plaque group (n = 5)	Yellow plaque group (n = 14)	Intensive yellow, ruptured plaquegroup (with ulceration and/or thrombus) (n = 6)	*P*
Age (years)	69 ± 11	68 ± 11	71 ± 10	0.820
Male [n (%)]	3 (60)	13 (92)	4 (67)	0.468
LAD/LCx/RCA [n (%)]	2 (40)/1 (20)/2 (40)	5 (36)/5 (36)/4 (28)	3 (50)/1 (17)/2 (33)	0.973
Body mass index	22.7 ± 4.0	24.1 ± 4.9	22.1 ± 3.1	0.620
Hypertension [n (%)]	3 (60)	12 (86)	3 (50)	0.213
Systolic blood pressure (mmHg)	127 ± 5	154 ± 23	138 ± 21	0.102
Diastolic blood pressure (mmHg)	74 ± 21	77 ± 9	69 ± 10	0.372
Current smoking [n (%)]	0 (0)	1 (7)	1 (17)	0.697
Dyslipidemia [n (%)]	2 (40)	10 (71)	2 (33)	0.227
LDL cholesterol (mg/dL)	68 ± 24	103 ± 45	117 ± 20	0.230
HDL cholesterol (mg/dL)	63 ± 17	62 ± 20	61 ± 12	0.917
Triglycerides (mg/dL)	109 ± 42	133 ± 85	165 ± 80	0.573
Total cholesterol (mg/dL)	154 ± 21	191 ± 50	211 ± 31	0.178
Diabetes [n (%)]	0 (0)	4 (29)	5 (83)	0.010
HbA1c (%)	5.2 ± 0.4	5.6 ± 0.9	7.7 ± 1.9*	0.002

Data are given as n (%) or mean ± SD

HDL: high-density lipoprotein, LDL: low-density lipoprotein

* *P* < 0.01 versus white plaque group and yellow plaque group

### Brachial-artery pulse wave velocity and level of high sensitivity C-reactive protein

**Fig. 2:**
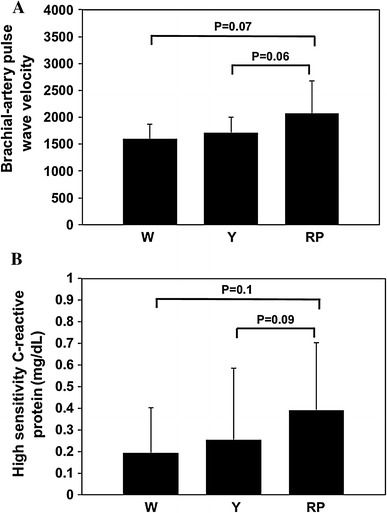
Brachial-artery pulse wave velocity (ba-PWV) and serum high-sensitivity C-reactive protein (hs-CRP) level: *Bar graphs* show ba-PWV (**A**) and the hs-CRP level (**B**) in the W-group (W), Y-group (Y), and RP-group (RP). ba-PWV and hs-CRP protein levels tended to be higher in the RP-group than in the other groups. Values represent mean ± SD

PWV is a useful clinical index for assessing cardiovascular risk and mortality [14, 15]. Moreover, plasma hs-CRP level is an important predictive factor for cardiovascular events [16]. Therefore, we studied PWV and hs-CRP in these groups. As shown in Fig. 2, ba-PWV and hs-CRP level tended to be higher in the RP-group than in the other groups, although the differences were not statistically significant (ba-PWV: W-group vs. RP-group, *P* = 0.07; Y-group vs. RP-group, *P* = 0.06; hs-CRP: W-group vs. RP-group, *P* = 0.1; and Y-group vs. RP-group, *P* = 0.09).

### Relationship between the angioscopic findings of the thoracic aorta and the coronary SYNTAX score

**Fig. 3:**
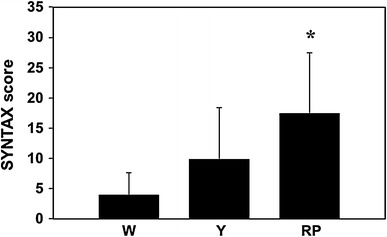
The extent and severity of coronary artery disease using SYNTAX score: A *bar graph* shows the SYNTAX score in the W-group (W), Y-group (Y), and RP-group (RP). SYNTAX score was significantly higher in the RP-group than in the W-group. *P < 0.05 versus W-group. Values represent mean ± SD

The SYNTAX score is a lesion-based angiographic grading system to determine the complexity of CAD [13]. The score is associated with adverse cardiovascular events and predicts mortality and morbidity in patients with CAD [17]. As shown in Fig. 3, the SYNTAX score was significantly higher in the RP-group than in the W-group (W-group 4.0 ± 3.6 vs. RP-group 17.5 ± 10.0, *P* = 0.045).

### Relationship between the angioscopic findings of the coronary artery and the thoracic aorta

**Fig. 4:**
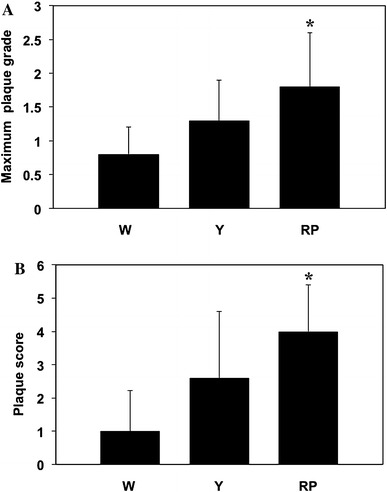
Maximum plaque grade and plaque score of the coronary artery: **A** a *bar graph* shows the maximum plaque grade of the coronary artery in the W-group (W), Y-group (Y) and RP-group (RP). Maximum plaque grade in the RP-group was significantly higher than that in the W-group, **B** a *bar graph* shows the plaque score of the coronary artery in the W-group (W), Y-group (Y) and RP-group (RP). Plaque score in the RP-group was significantly higher than in the W-group. *P < 0.05 versus W-group. Values represent mean ± SD

**Fig. 5:**
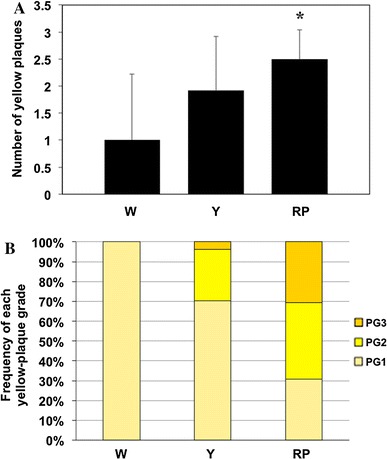
Number of yellow plaques in the coronary artery and frequency of yellow plaque grades in the groups: **A** a *bar graph* shows the number of yellow plaques in the observed coronary artery of W-group (W), Y-group (Y), and RP-group (RP). Number of yellow plaques in the RP-group was significantly higher than that in the W-group. *P < 0.05 versus W-group. Values represent mean ± SD, **B** a *bar graph* shows the frequency of each yellow-plaque grade in the W-group (W), Y-group (Y), and RP-group (RP). The yellow-plaque grade in a coronary artery was significantly correlated with the plaque grading of TA (*P* = 0.043). PG: plaque grade

The maximum plaque grade in the RP-group was significantly higher than in the W-group (W-group 0.8 ± 0.4 vs. RP-group 1.8 ± 0.8, *P* = 0.026; Fig. 4A). In addition, the plaque score in the RP-group was significantly higher than in the W-group (W-group 1.0 ± 1.2 vs. RP-group 4.0 ± 1.4, *P* = 0.014; Fig. 4B). Moreover, the number of yellow plaques in the observed coronary arteries and the frequency of the yellow-plaque grades in the groups were evaluated. The number of yellow plaques in the coronary arteries of RP-group was significantly higher than in the W-group (W-group 1.0 ± 1.2 vs. RP-group 2.5 ± 0.5, *P* = 0.023; Fig. 5A). In addition, the yellow-plaque grade in a coronary artery was significantly correlated with the plaque grading of TA (*P* = 0.043; Fig. 5B).

## Discussion

There were several major findings of this study. The angioscopic activity of the TA correlated well with that of the coronary artery and the severity of CAD. The angioscopic plaque grade, plaque score, and number of yellow plaques were significantly higher in patients with advanced atherosclerosis in the TA. In addition, the prevalence of diabetes was higher in the RP-group than in the other groups. The hs-CRP, an index of systemic inflammation, and ba-PWV, an index of arterial distensibility, tended to be correlated with the angioscopic activity of the TA and the coronary artery. According to the SYNTAX score, the severity of CAD was significantly correlated with the angioscopic atherosclerosis grade of the TA. To our knowledge, this is the first study to demonstrate that the plaque instability of the TA was intimately linked with coronary plaque vulnerability and severity. The angioscopic vulnerability of the TA and the coronary plaque was similar.

In the present study, 25 patients were evaluated according to the angioscopic grading of the TA. In a previous angioscopic study, diabetes mellitus was shown to be an independent clinical risk factor for silent plaque disruption in non-ischemic related coronary arteries [18]. Consistent with this report, the RP-group had a high prevalence of diabetes and high HbA1c levels. Plaque vulnerability was closely linked with high blood glucose levels on both the TA and the coronary artery angioscopy. From an angioscopic view, this suggests that systemic atherosclerosis and plaque vulnerability are more advanced in patients with diabetes.

Atherosclerosis is a generalized process that begins with medium-sized and large-sized vessels. Several studies have demonstrated a correlation of atherosclerosis between the TA and the coronary artery. Gu et al. using transesophageal echocardiography, reported that complex plaque in the TA was strongly associated with CAD [19]. In addition, Rohni et al. [11] demonstrated that the extent of atherosclerosis in the TA correlated with carotid IMT and the extent of CAD, which is consistent with current study findings; this suggests a close correlation between the degree of atherosclerosis in the TA and the coronary artery. In some autopsy studies, the prevalence of atherosclerotic plaques in the aorta has been studied in adults with CAD or its risk factors [20, 21]. Another study using MRI has indicated that the total extent of atherosclerosis in the aorta was closely related to the presence and extent of coronary stenosis [22]. Khoury et al. [23] demonstrated that the relationship between atherosclerotic disease in non-coronary arteries and the presence of CAD showed that extra-coronary plaques were a stronger predictor of CAD than conventional risk factors. In addition, a recent large population-based cohort study found an association between coronary artery calcification and descending TA calcification detected by cardiac CT; TA calcification was found to be a strong predictor of coronary artery calcification and might be an independent marker of risk for cardiovascular events [24]. In the current angioscopic study, the number of yellow plaques in the observed coronary artery was higher in the RP-group than in the W-group. Interestingly, the number of plaques in the coronary artery of the RP-group was similar to that of the Y-group; however, the angioscopic plaque vulnerability was higher in the RP-group than the Y-group. Diabetes or the presence of a systemic proinflammatory/proatherosclerotic environment in the patients of the RP-group might have affected the findings. Further studies will be necessary to explore these possibilities.

A number of studies have investigated whether ba-PWV is related to the presence of CAD. Cwynar et al. [25] showed that patients with CAD had higher ba-PWV, suggesting that high PWV could act as a predictor of established, widespread, atherosclerosis possibly including the coronary arteries. Similarly, a study using multidetector CT coronary angiography demonstrated that the mean ba-PWV was significantly higher in patients with coronary stenosis than in those without stenosis [26]. In the current study, the angioscopic degree of the atherosclerotic process was similar between the TA and the coronary artery while ba-PWV tended to be higher in the patients with angioscopically-advanced atherosclerotic lesions in the TA. These data suggested that arterial stiffness might increase along with the development of vulnerable plaque in the TA and the coronary artery.

The hs-CRP level, a strong risk predictor of cardiovascular event, tended to be higher in the RP-group than in the other groups. Several studies have reported that PWV was associated with increased hs-CRP level [27–29]. In addition, previous angioscopic studies reported that the intimal and serum CRP levels affected plaque characteristics and activity [30, 31]. Momiyama et al. [32] showed that CRP levels tended to reflect the severity of aortic atherosclerosis rather than coronary atherosclerosis. Consistent with these reports, the current study showed that the hs-CRP level tended to be higher in patients with high yellow-grade aortic atherosclerosis than in those with low yellow-grade aortic atherosclerosis. Taken together, the data suggest that the severity of plaque vulnerability might be correlated with arterial stiffness and CRP level, and that systemic inflammation may reflect an abnormal aortic wall.

As mentioned above, there are many studies investigating the relationship between the extent of atherosclerosis in the aorta and the presence and extent of coronary stenosis [11, 19, 22, 23]. On the other hand, data concerning the relationship of plaque vulnerability between the TA and the coronary artery have been lacking thus far. Here, the maximum yellow intensity of the coronary plaque was higher in the group with the highest atherosclerotic plaque activity in the TA than in the groups with low or moderate atherosclerotic plaque activity in the TA. In addition, the development of pan-coronary atherosclerosis expressed by the plaque score, number of yellow plaques, and frequency of the yellow-plaque grades were also higher in the patients with the highest atherosclerotic plaque activity in the TA. A clear and significant correlation between surface changes in the TA wall and in the coronary artery wall was also seen, suggesting that vulnerable plaques evolve simultaneously in both the systemic and coronary arterial tree. In addition, parameters such as aortic wall thickness, plaque morphologies, and vascular surface irregularities are strong predictors of future vascular events [33–35]. Current angioscopic techniques allow direct visualization and analysis of the TA wall plaque morphology, including color. These techniques provide more detailed diagnostic information than other modalities and might also serve as a predictor of future vascular events and a marker of the efficacy of anti-atherosclerotic treatments. However, further studies are required to answer this question.

This study had several limitations. First, the small number of patients was the main limitation of this study. Second, angioscopy cannot evaluate the entire vascular surface of the coronary artery and the TA. Third, some selection bias is inevitable, beginning with the selection of patients who are chosen to undergo cardiac catheterization and angioscopy.

In summary, the present study suggests that the angioscopic progression of aortic atherosclerosis might be closely associated with vulnerability to and the extent of coronary stenosis. The plaque instability of the TA was intimately linked with coronary plaque vulnerability and severity, indicating that vulnerability toward atherosclerotic plaque development occurs simultaneously in the coronary tree and systemic arteries.

## Electronic supplementary material

Supplementary material 1 (mov 388 kb)

Supplementary material 2 (mov 1153 kb)

Supplementary material 3 (mov 2736 kb)

Supplementary material 4 (mov 2558 kb)

## Abbreviations

TA: Thoracic aorta
CAD: Coronary artery disease
IMT: Intima-media thickness
W-group: White plaque group
Y-group: Yellow plaque group
RP-group: Intensive yellow, ruptured plaque with ulceration and/or thrombus group
ba-PWV: Brachial-artery pulse wave velocity
hs-CRP: High-sensitivity C-reactive protein
CT: Computed tomography
MRI: Magnetic resonance imaging
